# Genome-wide identification of GRF transcription factors in soybean and expression analysis of *GmGRF* family under shade stress

**DOI:** 10.1186/s12870-019-1861-4

**Published:** 2019-06-21

**Authors:** Feng Chen, Yingzeng Yang, Xiaofeng Luo, Wenguan Zhou, Yujia Dai, Chuan Zheng, Weiguo Liu, Wenyu Yang, Kai Shu

**Affiliations:** 1 Center for Ecological and Environmental Sciences, Northwestern Polytechnical University, Xi’an, 710129 China; 2 Institute of Ecological Agriculture, Sichuan Agricultural University, Chengdu, 611130 China

**Keywords:** Growth-regulating factor, Gene family, Shade, Leaf size, Soybean, Seed

## Abstract

**Background:**

The *Growth*-*regulating factor* (*GRF*) family encodes plant-specific transcription factors which contain two conserved domains, QLQ and WRC. Members of this family play vital roles in plant development and stress response processes. Although *GRFs* have been identified in various plant species, we still know little about the *GRF* family in soybean (*Glycine max*).

**Results:**

In the present study, 22 *GmGRFs* distributed on 14 chromosomes and one scaffold were identified by searching soybean genome database and were clustered into five subgroups according to their phylogenetic relationships. *GmGRFs* belonging to the same subgroup shared a similar motif composition and gene structure. Synteny analysis revealed that large-scale duplications played key roles in the expansion of the *GmGRF* family. Tissue-specific expression data showed that *GmGRFs* were strongly expressed in growing tissues, including the shoot apical meristems, developing seeds and flowers, indicating that *GmGRFs* play critical roles in plant growth and development. On the basis of expression analysis of *GmGRFs* under shade conditions, we found that all *GmGRFs* responded to shade stress. Most *GmGRFs* were down-regulated in soybean leaves after shade treatment.

**Conclusions:**

Taken together, this research systematically analyzed the characterization of the *GmGRF* family and its primary roles in soybean development and shade stress response. Further studies of the function of the *GmGRFs* in the growth, development and stress tolerance of soybean, especially under shade stress, will be valuable.

**Electronic supplementary material:**

The online version of this article (10.1186/s12870-019-1861-4) contains supplementary material, which is available to authorized users.

## Background

Growth-regulating factors (GRFs) are plant-specific transcription factors which regulate plant growth, development and abiotic stress response [1–5]. The first *GRF*, named *OsGRF1*, was identified from deepwater rice (*Oryza sativa*); expression of *OsGRF1* was induced by gibberellin (GA), and it mediated stem elongation in a GA-dependent manner [6]. Numerous studies demonstrated that there are two conserved domains, QLQ and WRC, in the N-terminal region of GRF proteins. The QLQ (Gln, Leu, Gln) domain serves as a protein-protein interaction feature which could interact with the GRF-interacting factor (GIF) [7], while the plant-specific WRC (Trp, Arg, Cys) domain comprises a C_3_H motif for DNA binding and a nuclear localization signal (NLS) [8]. Compared with the conserved nature of the amino acid residue sequence in the N-terminal region, the C-terminal region of the GRFs is variable, with some investigations indicating that the C-terminal region possessed transactivation activity [7–9]. In addition, some less-conserved motifs, such as TQL and FFD, are usually present in the C- terminal region of GRFs [10].

As they are development-related transcription factors, it is not strange that GRFs mediate the shape and size of leaves by regulating cell proliferation [11, 12]. Overexpression of *AtGRF1*, *AtGRF2* and *AtGRF5* resulted in larger leaves than in wild-type (WT) *Arabidopsis*, while the leaves of *grf* mutants, such as *grf3–1*, *grf5–1*, *grf1–1/grf2*, *grf2/grf3* and *grf1/2/3*, were much smaller than the WT [1, 2, 11, 12]. Furthermore, *GRFs* also regulate root growth, floral development and seed size [9, 13–15]. In addition to GRF, the GIFs, are also involved in the control of leaf size and architecture. Compared to the WT, the leaves of *gif1* were narrower and smaller [7, 16]. Biochemical analysis showed that GRF and GIF combine to form a transcriptional complex in vivo to modulate cell proliferation and ultimately to control leaf size [5, 16]. Furthermore, it has been shown that the microRNA miR396 directly inhibits the expression of *GRFs* through post-transcriptional regulation [11, 14]. Constitutive overexpression of *Arabidopsis miR396a* and *miR396b*, or heterologous expression of *ptc-miR396c* (from *Populus trichocarpa*) and *ath-miR396a* (from *Arabidopsis thaliana*) in tobacco (*Nicotiana tabacum*) significantly reduce mRNA levels of *GRFs* and lead to narrower and smaller leaves, which mimic *Arabidopsis grf1/2/3* [17–20]. Thus, the transcription levels of *GRFs* are regulated strictly and quantitatively by the miRNA-GRF-GIF cascade.

Light is an important environmental factor that plays critical roles in plant growth and development, and ultimately determines crop yield [21]. However, in maize (*Zea mays*)-soybean (*Glycine max*) relay strip intercropping systems, the light environment in the soybean canopy changes due to it being lower than the maize canopy, so that soybean was under shade stress (resulting from reductions in light quantity and in the red: far-red light ratio) [22, 23]. Furthermore, dense-planting patterns in crops also lead to shade stress among neighboring seedlings [24]. Previous studies had demonstrated that soybean morphological traits changed markedly under shade conditions, resulting in increased plant height, decreased yield, and reduced root length [23, 25, 26]. Notably, leaf expansion is also suppressed when soybean responds to shade stress [27, 28]. However, the specific regulatory mechanisms underlying leaf development under shade conditions are still largely unknown, especially with respect to the *GRF* family-mediated pathways.

In the present study, 22 *GmGRFs* were dissected from the soybean genome. Their sequence characteristics, chromosome distribution, phylogenetic relationships, gene structures, conserved motif compositions and synteny were then systematically characterized. Based on these findings, the expression profiles of *GmGRFs* in various vegetative and reproductive tissues were documented. Furthermore, we also analyzed the expression patterns of *GmGRFs* under shade stress conditions. These results will not only help us to better understand the functions of the *GmGRF* family, but will also provide a foundation for improving crops, especially soybean, through genetic modification.

## Results

### Identification of GmGRFs

Based on the Hidden Markov Model (HMM) of WRC and QLQ domains, a total of 22 *GmGRFs* were identified from soybean genome and named as *GmGRF1* – *GmGRF22* according to their locations on chromosomes (Table 1). The length of the coding sequences (CDS) of *GmGRFs* varied from 927 bp (*GmGRF10*) to 1830 bp (*GmGRF19*). Accordingly, GmGRF10, with 308 amino acid residues, was the smallest GmGRF, whereas the largest GmGRF was GmGRF19 (609 amino acid residues). The theoretical molecular weight (MW) of these putative GmGRFs ranged from 34.58 to 66.90 kDa and the isoelectric point (pI) ranged from 6.35 (GmGRF13) to 9.10 (GmGRF1) (Table 1).

**Table 1:** Characterization of the *GmGRF* family in soybean

Serial No.	Name	Gene	CDS (bp)	Length (aa)	MW (Da)	pI
1	*GmGRF1*	Glyma.01G148600	1008	335	36,936.17	9.10
2	*GmGRF2*	Glyma.01G234400	957	318	35,503.27	7.76
3	*GmGRF3*	Glyma.03G192200	1155	384	41,814.42	7.00
4	*GmGRF4*	Glyma.04G230600	1782	593	63,535.80	8.31
5	*GmGRF5*	Glyma.06G134600	1737	578	62,125.92	8.88
6	*GmGRF6*	Glyma.07G038400	1038	345	39,463.48	8.51
7	*GmGRF7*	Glyma.09G068700	1098	365	41,534.98	8.31
8	*GmGRF8*	Glyma.09G212500	1014	337	37,342.63	8.99
9	*GmGRF9*	Glyma.10G067200	1011	336	37,307.55	7.16
10	*GmGRF10*	Glyma.11G008500	927	308	34,583.24	7.76
11	*GmGRF11*	Glyma.11G110700	1026	341	38,021.95	7.26
12	*GmGRF12*	Glyma.11G208800	1008	335	36,722.82	6.49
13	*GmGRF13*	Glyma.12G014700	1002	333	36,915.65	6.35
14	*GmGRF14*	Glyma.13G109500	1155	384	43,467.94	8.53
15	*GmGRF15*	Glyma.15G176500	1098	365	41,453.74	8.08
16	*GmGRF16*	Glyma.16G007600	1083	360	40,950.97	8.51
17	*GmGRF17*	Glyma.17G050200	1134	377	42,704.24	8.08
18	*GmGRF18*	Glyma.17G232600	1803	600	65,630.47	7.75
19	*GmGRF19*	Glyma.17G232700	1830	609	66,897.44	6.70
20	*GmGRF20*	Glyma.19G192700	1167	388	42,108.80	6.63
21	*GmGRF21*	Glyma.U028600	1800	599	65,601.45	7.20
22	*GmGRF22*	Glyma.U028700	1785	594	64,900.59	6.52

**Fig. 1:**
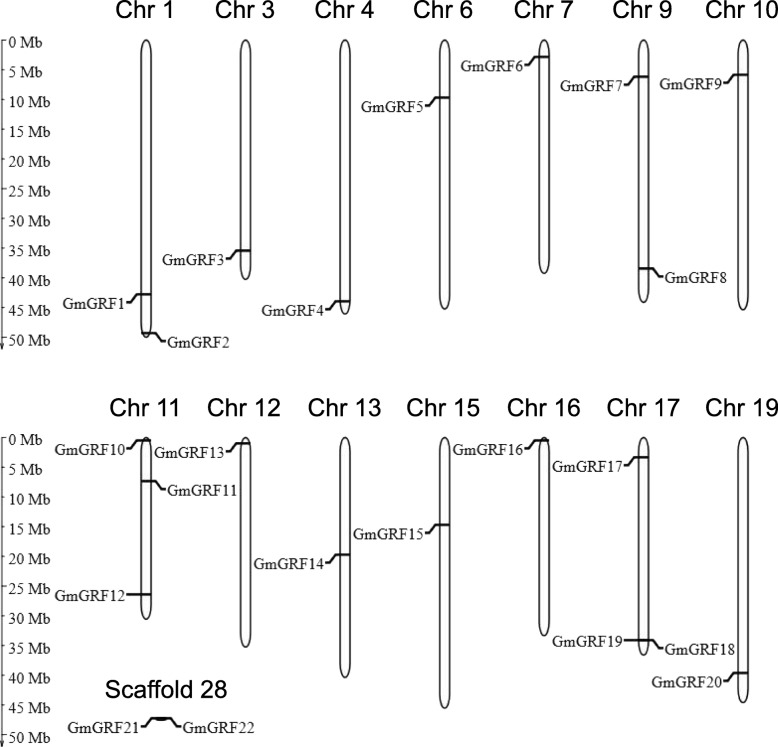
Distribution of *GmGRFs* on soybean chromosomes or scaffold

*GmGRFs* were unevenly distributed across the chromosomes. Twenty *GmGRFs* were distributed on 14 chromosomes and two *GmGRFs* were located on scaffold_28 (Fig.1). Of the 14 chromosomes, chromosomes 11 and 17 contained the largest number of *GmGRFs*, each with three genes. Both chromosomes 1 and 9 carried two *GmGRFs* each, while only one *GmGRF* was observed on each of chromosomes 3, 4, 6, 7, 10, 12, 13, 15, 16 and 19 (Fig.1).

### Sequence and phylogenetic analysis of GmGRFs

**Fig. 2:**
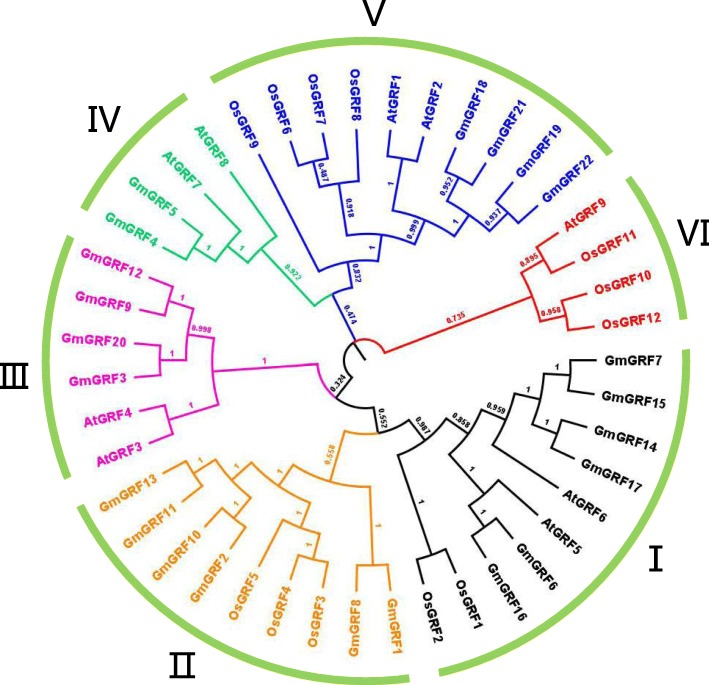
Phylogenetic analysis of GRFs from *Glycine max* (Gm), *Oryza sativa* (Os) and *Arabidopsis thaliana* (At). Clustal W was used to align 43 GRFs, namely nine AtGRF, 12 OsGRF, and 22 GmGRF, while MEGA 7.0 software was employed to construct a neighbor-joining phylogenetic tree with 1000 bootstrap replications

All GmGRFs contained conserved QLQ and WRC domains in their N-terminal regions (Additional file 1: Figure S1). To gain an insight into the evolutionary relationships among GRFs from soybean (22), rice (12) and *Arabidopsis* (9), MEGA 7.0 software was used to construct a neighbor-joining phylogenetic tree. As shown in Fig. 2, a total of 43 GRFs from different species were clustered into six subgroups (I–VI). Five of the six subgroups contained GmGRFs, whereas subgroup VI had only AtGRF and OsGRF members. Among the six subgroups, subgroups IV and VI were relatively small, with only four GRFs each. By contrast, subgroups I and V contained the largest number of GRFs (ten each), followed by subgroups II (nine), III (six). The phylogenetic tree suggested that the GmGRFs showed a closer relationship with AtGRFs than with OsGRFs, which may be partly because both soybean and *Arabidopsis* are dicotyledonous plants.

### Structural analysis of *GmGRFs*

**Fig. 3:**
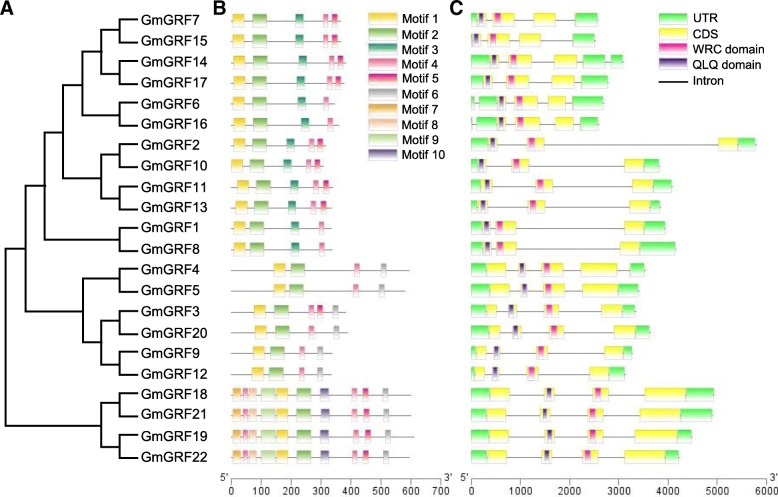
Phylogenetic analysis, gene structures and conserved motifs of *GmGRFs*. **a** The phylogenetic relationship of *GmGRFs*. A neighbor-joining phylogenetic tree was constructed by MEGA7.0 software with the Poisson model and 1000 bootstrap replications. **b** Conserved motif arrangements of GmGRFs. Ten conserved motifs labeled with different colors were found in the GmGRF sequences using the MEME program. Among them, motif 1 and motif 2 are the QLQ and WRC conserved domains. **c** Exon-intron organizations of *GmGRFs*. The green boxes represent 5’or 3′ untranslated regions, yellow boxes represent the coding sequences, and black lines represent the introns. The lengths of the exons and introns can be determined by the scale at the bottom

To further explore the evolutionary relationship among GmGRFs, we constructed a phylogenetic tree and analyzed the gene structures and motif characteristics of *GmGRFs* (Fig. 3). According to Fig. 2, GmGRFs could be clustered into five groups. It was obvious that the 22 *GmGRFs* contained two to four introns (six genes with two introns, twelve with three introns, and four with four introns) (Fig. 3c). The conserved structure of *GmGRFs* was similar to those from other plant species, in which most genes contained three introns [29–31]. The lengths of individual *GmGRFs* were variable in intron length could partly reflect the length of different genes. For instance, the longest gene, *GmGRF2*, with a size of 5.7 kb, was due mainly to the fact that it contained a total intron length of 4.8 kb.

The Multiple EM for Motif Elicitation (MEME) web server was employed to identify the conserved motifs of GmGRFs (Fig. 3b and Additional file 2: Table S1). All GmGRFs contained motif 1 and motif 2 which were annotated as the GRF specific domains, QLQ and WRC, in their N-terminal regions. Each GmGRF has between four and ten conserved motifs, with the GRFs of subgroup V (GmGRF18, GmGRF19, GmGRF21 and GmGRF22) containing the largest number of motifs. The GmGRFs belonging to the same subgroup have a similar motif composition (e.g. GmGRF4 and GmGRF5, GmGRF18 and GmGRF21). In addition, some motifs appeared in only certain specific subgroups. For example, motif 3 is unique to subgroup I and II, while motifs 7, 8, 9 and 10 are specific to subgroup V. Overall, gene structures and motif characteristics strongly supported the phylogenetic relationships of GmGRFs.

### Synteny analysis of *GmGRFs*

**Fig. 4:**
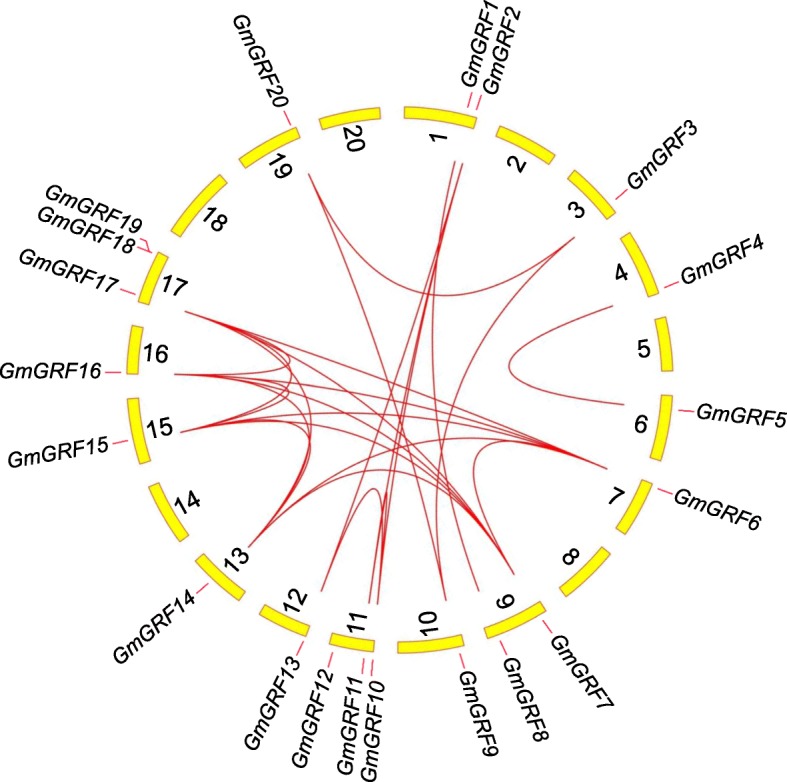
The syntenic relationships among *GmGRFs*. The scaffold_28 was not shown in the Circos map

Gene duplication plays an important role in increasing the numbers of genes and their subsequent evolution. For instance, over 90% of regulatory and developmental genes in the *Arabidopsis* genome had been duplicated [32]. In order to analyze the duplication events of *GmGRFs*, Multiple Collinearity Scan toolkit X (MCScanX) software was employed. Ultimately, we found that 19 of the 22 *GmGRFs* (86.36%) were distributed in the duplication regions, suggesting that these genes were generated by large-scale duplication events, whole-genome duplication (WGD) or segmental duplication (Fig. 4 and Additional file 3: Table S2). Additionally, according to a previous methodology [33], *GmGRF18*-*GmGRF19* and *GmGRF21*-*GmGRF22* belonging to chromosome 17 and scaffold_28, respectively, were identified as tandem duplication genes. Further, we calculated the nonsynonymous substitution rate (Ka) and synonymous substitution rate (Ks) of these duplicated gene pairs. The results showed that the Ka/Ks ratios of most *GmGRF* pairs were less than 1, indicating that these *GmGRFs* had undergone purifying selection processes.

### Expression profiles of *GmGRFs*

**Fig. 5:**
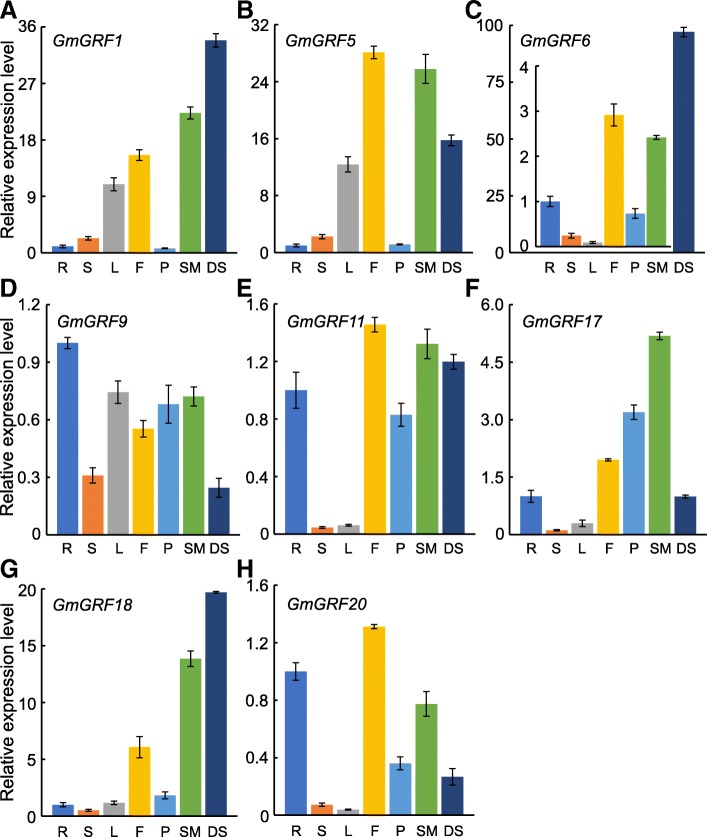
Expression profiles of *GmGRFs* in seven tissues. Expression levels of eight selected *GmGRFs* were examined by qRT-PCR. The housekeeping *GmTubulin* was used as an endogenous reference gene. R, root; S, stem; L, leaf; F, flower; P, pod; SM, shoot apical meristem; DS, developing seed. Error bars represent standard errors

Based on the analysis of the results from Figs. 2, 3 and 4, we selected eight *GmGRFs* belonging to different subgroups for GA response and tissue-specific expression analysis. After GA_3_ treatment, the expression levels of all selected eight *GmGRFs* were down-regulated (Additional file 4: Figure S2). Then we investigated the expression patterns of these *GmGRFs* in seven soybean tissues (roots, flowers, stems, pods, leaves, shoot apical meristem, and developing seeds). As shown in Fig. 5, although all *GmGRFs* were expressed in all seven tissues, the transcription levels of the different genes varied greatly among the different tissues. In general, *GmGRFs* were highly expressed in growing tissues, such as developing seeds, flowers and shoot apical meristems. Of the *GmGRFs*, the expression levels of *GmGRF1*, *GmGRF6* and *GmGRF18* were highest in developing seeds (Fig. 5a, c and g). Among these three genes, *GmGRF6* was more preferentially expressed in developing seeds, indicating 100-fold higher than that in root. However, in flowers, the transcription levels of *GmGRF5*, *GmGRF11* and *GmGRF20* were highest (Fig. 5b, e and h). Furthermore, *GmGRF9* was abundantly expressed in roots, while *GmGRF17* showed the highest transcription level in shoot apical meristems (Fig. 5d and f). The results demonstrated multiple potential functions of *GmGRFs* in regulating growth and development of distinct soybean tissues.

### Expression profiles of *GmGRFs* in response to shade stress

**Fig. 6:**
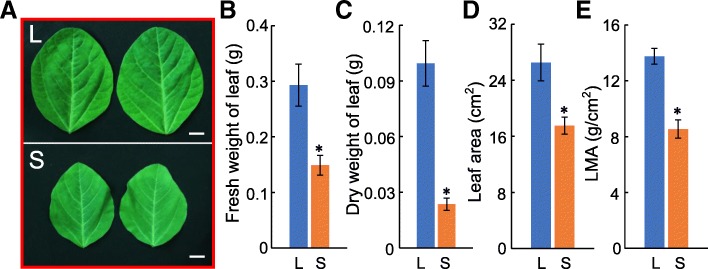
Soybean leaf area and weight decreased under shade conditions. **a** Representative photographs of soybean leaves under white light and shade conditions. Bar = 10 mm. Leaf fresh and dry weight (**b**, **c**), leaf area (**d**) and leaf mass per area (LMA; **e**) were analyzed after shade stress. Ten soybean leaves were measured under each condition. Error bars represent standard errors. The asterisk (^*^) indicates the significant difference at *P <* 0.05 by Student’s *t*-test analysis. L, shade; S, shade

**Fig. 7:**
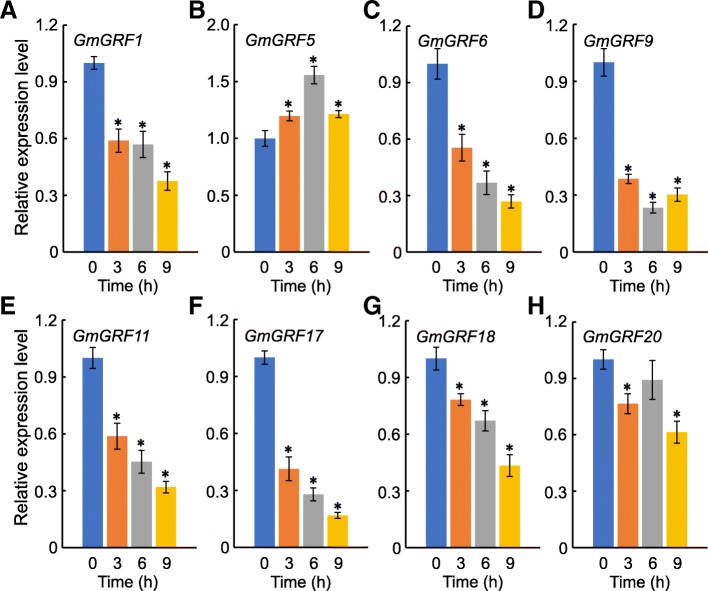
Time course of expression patterns of *GmGRFs* under shade conditions. The housekeeping *GmTubulin* was used as an internal control. Error bars represent standard errors. The asterisk (^*^) indicates the significant difference at *P <* 0.05 by Student’s *t*-test analysis

Soybean plant morphology changed greatly in response to shade stress, dubbed as shade avoidance syndrome (SAS), including decreased leaf area and weight, and excessive elongation of stems (Fig. 6 and Additional file 5: Figure S3). Previous studies have demonstrated that *GRFs* play important roles in regulating leaf size and plant response to abiotic stresses, including abscisic acid (ABA) and osmotic stresses [4, 34–36]. Therefore, in order to further explore the roles of *GmGRFs* under shade conditions, we measured the transcription levels of eight *GmGRFs* by qRT-PCR. The results showed that the transcription of all the *GmGRFs* tested was influenced by shade. Expression of almost all *GmGRF*s was significantly down-regulated under shade stress, albeit to different degrees (Fig. 7). Among these genes, the transcription levels of *GmGRF9* and *GmGRF17* were more strongly suppressed, while *GmGRF20* showed the weakest transcription repression. On the other hand, the expression level of *GmGRF5* was up-regulated under shade stress conditions (Fig. 7b). Based on the expression patterns of *GmGRFs*, *GmGRF5*, *GmGRF9* and *GmGRF17* may play important roles in regulating leaf growth under shade conditions.

## Discussion

As members of plant-specific gene family, *GRFs* play important roles in plant growth and development, especially in regulating organ size [5, 11, 15]. Recent studies in rice have shown that *OsGRF4* can improve rice yield by promoting cell division and nitrogen uptake efficiency [37–39]. Therefore, it is necessary to understand the mechanisms regulating levels of GRFs during plant development and response to stress. To date, *GRF* family has been identified in various plants (Additional file 6: Table S3), such as *Arabidopsis*, rice, *Zea mays*, *Brassica napus*, *Citrus sinensis*, *Camellia sinensis*, *Populus trichocarpa* and *Pyrus×bretschneideri* [1, 8, 40–43]. However, little information on the function of GRFs in soybean is available.

In order to better understand the characteristics and functions of *GmGRFs*, we studied members of the *GmGRF* family by bioinformatics assay, qRT-PCR assay, and plant morphology analysis. In the current study, a total of 22 *GmGRFs* was identified by searching soybean genome database, and all putative GmGRFs were shown to contain QLQ and WRC domains (Table 1 and Additional file 1: Figure S1). Phylogenetic analysis showed that 22 *GmGRFs* could be clustered into five subgroups according to their evolutionary relationships (Fig. 1). The exon-intron organizations and motif arrangements were consistent with the phylogenetic analysis, while the structure of *GmGRFs* belonging to different subgroups displayed lower identities.

Numerous studies have shown that gene duplication can not only increase the number of *GRFs*, but is also an avenue for generating novel genes, a phenomenon which is conducive to enabling the plant to adapt to various environments [44–47]. Indeed, the expansion of the *GRF* family occurred mainly through gene duplication, especially large-scale duplications (WGD or segmental duplications) [5, 30, 43]. Consistently, in the present study, most *GmGRFs* were distributed in duplication blocks, indicating that WGD or segmental duplications had played major roles in the expansion of the *GmGRF* family. In addition, soybean contains more *GRFs* than does another legume, *Medicago truncatula*, a model plant (Additional file 6: Table S3 and Additional file 7: Table S4). This may mainly be due to two WGD events which occurred during the evolution of the soybean genome (58 and 13 million years ago), while the *Medicago* genome experienced only a single WGD event at 58 million years ago [48, 49]. It is noted that this phenomenon also exists in other soybean gene families, such as the homeodomain-leucine zipper and homeobox gene families [50, 51]. Altogether, these results suggested that large-scale duplications were universal during the expansion process of the *GmGRF* family.

Previous research had reported that *GRFs* are pivotal regulators of plant growth and development [5, 34]. For example, overexpression of *AtGRF5* leads to leaf enlargement, while the leaves of the *Arabidopsis grf5* mutant are narrower than those of the WT [12]. Generally, the expression levels of *GRFs* in actively growing tissues was higher than that in mature tissues [29]. Indeed, several studies had revealed that, with the aging of organs, the transcription levels of *GRFs* decreased [19, 41]. In the present study, we found that *GmGRFs* are highly expressed in shoot apical meristems, developing seeds and flowers (Fig. 5). Interestingly, among all eight selected *GmGRFs*, *GmGRF1*, *GmGRF6* and *GmGRF18* were highly expressed in developing seeds, suggesting that these genes may play important roles in seed development. In addition to developing seeds, *GRFs* are also important in flower development and root growth [9, 52–54]. Correspondingly, we found that some *GmGRFs*, such as *GmGRF9*, *GmGRF11*and *GmGRF20*, were expressed abundantly in flowers and roots. The gene expression analysis indicated that GmGRFs may play important roles in the growth and development of soybean tissues.

Light not only provides energy for plants, but also regulates plant growth and development [55, 56]. Plants can sense changes in the light environment through photoreceptor systems, such as phytochromes, cryptochromes and phototropins [57–59]. As sessile photoautotrophs, plants need to compete with neighbors for light, nutrients and other resources. For example, when exposed to shade stress from neighboring plants, plants adapted to open ranges (e.g. *Arabidopsis*, rice and soybean) can develop shade avoidance response, leading to shade avoidance syndrome [60–62]. Shade avoidance response is conducive to survival of the plant under shade conditions [61, 63]. In the context of agricultural production, however, shade avoidance can cause a decline in crop yield [63]. In maize-soybean intercropping systems, for example, soybean yield decreased significantly, because of the shade stress arising from the taller neighboring maize plants [23]. In the present investigation, the transcription of almost all *GmGRF* genes tested decreased under shade treatment (Fig. 7), suggesting that *GmGRFs* possess the veiled functions with regard to plant shade response.

The leaf is the primary tissue of photosynthesis and its size directly affects photosynthetic efficiency [64, 65]. Numerous studies have shown that leaf area is regulated by many genes related to cell division and expansion [36]. Among them, *GRFs* can regulate leaf area by controlling cell proliferation [65]. In addition, UV-B radiation inhibits leaf growth by decreasing the expression of *GRF*s in *Arabidopsis* and maize [66, 67]. Morphological analysis showed that soybean leaf size, leaf area and dry and fresh weight decreased significantly under shade stress (Fig. 6), and, in line with this, the expression levels of almost all *GmGRF* genes were down-regulated under shade conditions (Fig. 7). Given the positive regulatory effect of GRFs on plant cell proliferation in diverse plant species [68], these results were consistent (Figs. 6 and 7). Interestingly, under shade conditions, the expression levels of most *GmGRFs* were down-regulated while *GmGRF5* transcription was up-regulated under these conditions. Therefore, further study of the function of *GmGRF5* under shade stress may be helpful in increasing the leaf area of intercropped soybean, thereby potentially increasing the yield of soybean.

## Conclusions

In this study, we systematically analyzed the basic characteristics and functions of *GmGRFs*. A total of 22 GmGRFs were identified from the soybean genome with the help of the HMM of two conserved domains specific to GRF, namely QLQ and WRC. These *GmGRFs* were distributed on 14 chromosomes and one scaffold, and could be clustered into five subgroups according to their phylogenetic relationships. By further analysis, we found that *GmGRFs* belonging to the same subgroup had similar gene structures and motif compositions. Gene duplication analysis suggested that both large-scale and tandem duplications, especially the former, contributed to the expansion of the *GmGRF* family. The result of qRT-PCR studies showed that *GmGRFs* were involved in the growth and development of soybean, as well as in shade response. Importantly, we identified some potentially useful genes, such as *GmGRF1*, *GmGRF5* and *GmGRF6* by analyzing the *GmGRF* family. These results will provide a foundation for future research on the *GRFs* in soybean.

## Methods

### Plant materials and shade treatment

Soybean cultivar Nandou-12, a prevailing cultivar in southwestern China, was employed in this study. The seeds of Nandou-12 were originally obtained from Nanchong Institute of Agricultural Sciences, Sichuan Province, China. Soybean plants were grown in a phytotron with 65% relative humidity, a 12-h light (25 °C)/12-h dark (20 °C) photoperiod and a light intensity of 380 μmol m^− 2^ s^− 1^. In order to analyze the expression patterns of *GmGRFs* in different tissues of soybean, we collected roots (16 d after sowing), stems (16 d after sowing), leaves (16 d after sowing), shoot apical meristems (16 d after sowing), flowers (36 d after sowing), pods (44 d after sowing) and developing seeds (60 d after sowing). For the GA treatment, six-day soybean seedlings were sprayed with 100 μM GA_3_ and the hypocotyls of soybean seedlings were harvested after 0, 3, 6 and 9 h. Furthermore, to explore the transcription profiles of *GmGRFs* under shade stress, black nylon net and far-red light-emitting diode (LED) were employed to adjust the light environment (light intensity and quality) [26]. 10-day soybean seedlings were transferred to the shade environment with 113 μmol m^− 2^ s^− 1^ of photosynthetically active radiation (PAR) and 0.4 of red: far-red light ratio (R/FR). The first compound leaves of soybean seedlings were collected at 0, 3, 6 and 9 h after shade treatment. All samples were frozen immediately in liquid nitrogen and then stored at − 80 °C for further analysis.

Seven days after shade treatment, ten soybean seedlings from different pots were used for analyzing morphological characteristics. Leaf area of first compound leaves was measured by ImageJ software. Seeding height, stem diameter, leaf and root fresh weight were also determined. Subsequently, leaves, stems and roots were exposed to 105 °C for 0.5 h and then dried at 80 °C until constant weight. Finally, dry weight, root-shoot ratio and leaf mass per area (LMA) were also calculated.

### Identification of *GmGRFs*

Soybean protein sequences and genome annotation were downloaded from Phytozome database (http://www.phytozome.net/). The HMM of WRC (PF08879) and QLQ (PF08880) domains were obtained from PFAM database (http://pfam.xfam.org/) and used to predict GmGRFs with HMMER software. Then, these protein sequences (E-value ≤1e^− 10^) were used to construct soybean-specific HMM for identifying GmGRFs [69]. Finally, predicted proteins were considered as GmGRFs only if they contained QLQ and WRC conserved domains verified by SMART software (http://smart.embl-heidelberg.de/) and PFAM databases. All putative *GmGRF*s were drawn on chromosomes using MapGene2Chrom web v2 (http://mg2c.iask.in/mg2c_v2.0/).

### Sequence and phylogenetic analysis

The exon-intron distribution patterns of *GmGRFs* were analyzed using Dual Systeny Plotter software (https://github.com/CJ-Chen/TBtools) [70, 71]. To predict MW and pI of GmGRFs, the ExPASy proteomics server (https://web.expasy.org/protparam/) was used [72]. Conserved motifs of GmGRFs were predicted by MEME online program (http://meme-suite.org/tools/meme) [73]. Multiple sequence alignments of GmGRFs were analyzed by Clustal W [74]. MEGA 7.0 software was employed to construct phylogenetic trees using the neighbor-joining method with Poisson model, pairwise deletion, and 1000 bootstrap replications [75].

### Gene duplication and evolution analysis

MCScanX program with the default parameters was used to analyze the duplication events of *GmGRFs* [76]. According to the results of MCScanX, the nonsynonymous substitution rate (Ka) and synonymous substitution rate (Ks) of duplicated genes were calculated by KaKs_Calculator 2.0 software [77].

### Gene expression analysis

Total RNA preparation, first-strand cDNA synthesis and the qRT-PCR assay were performed as previously described [78]. According to the manufacturer’s protocol, total RNA was treated with DNase I and then 2 μg total RNA was reverse-transcribed using Moloney murine leukemia virus reverse transcriptase (200 units per reaction; Promega Corporation). The qRT-PCR was performed using Vazyme™ AceQ qPCR SYBR Green Master mix on a QuantStudio 6 Flex Real-Time PCR System (Thermo Fisher Scientific, USA) [79]. The soybean housekeeping *GmTubulin* was used as an endogenous reference gene and each reaction had three repetitions. 10 μL reaction mixture included 5 μL Vazyme™ AceQ qPCR SYBR Green Master mix, 0.2 μL forward primer, 0.2 μL reverse primer, 1 μL cDNA template and 3.6 μL Dnase-free ddH_2_O. The qRT-PCR reaction procedure was set as follows: 95 °C for 30s, and then 40 cycles of 95 °C for 15 s, 60 °C for 30 s. The expression levels of *GmGRFs* were calculated by the comparative CT method [80]. Sequences of the primers for qRT-PCR were listed in Additional file 8: Table S5.

### Statistical analysis

In this study, the Student’s *t*-test in Microsoft Excel software was used to analyze the differences between different treatments. *P* < 0.05 was considered as statistical significance.

## Additional files

Additional file 1: **Figure S1.** Sequence alignment of two conserved domains, QLQ and WRC, of the GmGRFs. (PDF 228 kb)

Additional file 2: **Table S1.** Characterization of ten conserved motifs in GmGRF sequences. (PDF 23 kb)

Additional file 3: **Table S2.** Duplication events of *GmGRFs*. (PDF 44 kb)

Additional file 4: **Figure S2.** The transcription levels of *GmGRFs* in response to GA_3_. The housekeeping *GmTubulin* was used as an internal control. Error bars represent standard errors. The asterisk (*) indicates the significant difference at P < 0.05 by Student’s *t*-test analysis. (PDF 46 kb)

Additional file 5: **Figure S3.** Shade stress induces shade avoidance response in soybean. (A) Representative photographs of soybean seedlings under white light and shade conditions. Bar = 100 mm. Seeding height (B), stem diameter (C), above-ground tissue fresh and dry weight (D, F), root fresh and dry weight (E, G) and root-shoot ratio (H). Ten soybean seedlings were measured under each condition. Error bars represent standard errors. The asterisk (^*^) indicates the significant difference at *P <* 0.05 by Student’s *t*-test analysis. L, shade; S, shade. (PDF 134 kb)

Additional file 6: **Table S3.** Genome size and *GRF* number in plants. (PDF 32 kb)

Additional file 7: **Table S4**. *MtGRFs* in *Medicago*. (PDF 20 kb)

Additional file 8: **Table S5.** Primers sequence used in this study. (PDF 40 kb)

## Data Availability

All data used and analyzed in this study are included in the article and its additional file.

## Abbreviations

ABA: Abscisic acid
GA: Gibberellin
GIF: GRF-interacting factor
GRF: Growth-regulating factor
HMM: Hidden Markov Model
Ka: Nonsynonymous substitution rate
Ks: Synonymous substitution rate
LED: Light-emitting diode
LMA: Leaf mass per area
MCScanX: Multiple Collinearity Scan toolkit X
MEME: Multiple EM for Motif Elicitation
MW: Molecular weight
NLS: Nuclear localization signal
PAR: Photosynthetically active radiation
pI: Isoelectric point
R/FR: Red: far-red light ratio
SAS: Shade avoidance syndrome
WGD: Whole-genome duplication
WT: Wild-type
