# Supplementary material for: Genome-wide identification of GRF transcription factors in soybean and expression analysis of GmGRF family under shade stress
Source: BMC Plant Biol. 2019 Jun 21;19:269. doi: 10.1186/s12870-019-1861-4 (PMC6588917; doi:10.1186/s12870-019-1861-4)

Additional file 1: Figure S1

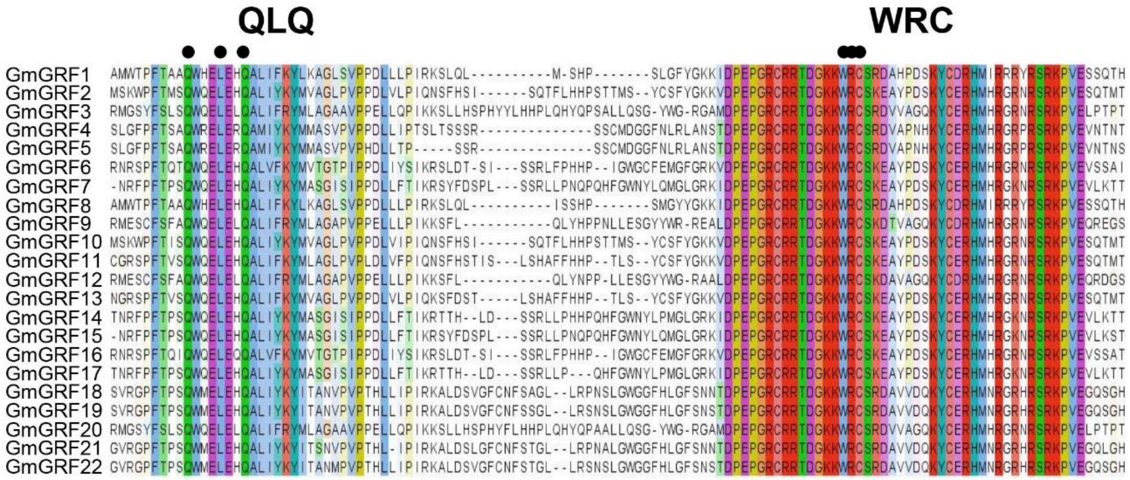

Supplement: Supplementary file 1 — Figure S1. Sequence alignment of two conserved domains, QLQ and WRC, of the GmGRFs. (PDF 228 kb) [file 12870_2019_1861_MOESM1_ESM.pdf]
