# Supplementary material for: Genome-wide identification of GRF transcription factors in soybean and expression analysis of GmGRF family under shade stress
Source: BMC Plant Biol. 2019 Jun 21;19:269. doi: 10.1186/s12870-019-1861-4 (PMC6588917; doi:10.1186/s12870-019-1861-4)
Supplement: Supplementary file 2 — Table S1. Characterization of ten conserved motifs in GmGRF sequences. (PDF 23 kb) [file 12870_2019_1861_MOESM2_ESM.pdf]

Additional file 2: Table S1. Characterization of ten conserved motifs in GmGRF sequences.

Motif	Width	E-value	Best possible match
**1**	41	1.3e-561	MRSPFTPSQWQELEHQALIYKYMVAGLPVPPDLLLPIKKSL
**2**	50	1.9e-940	GKKVDPEPGRCRRTDGKKWRCSKDAYPDSKYCERHMHRG RNRSRKPVEVQ
**3**	29	2.2e-096	RYVYGLKSEVDEHAFFTEPLGSMKVLSAE
**4**	20	1.8e-123	LKZEQHSLRHFFDEWPKSKR
**5**	21	1.1e-085	KSSTTQLSISIPISSSDFATF
**6**	21	2.7e-060	IPITWESSMGGPLAEVLRSST
**7**	29	4.7e-051	GLEGVVGSESGCVFGSSLASDPETKHKWY
**8**	29	1.5e-040	AKTDHDMSSASKAMLFQQRNNSLLRSNNA
**9**	50	7.3e-061	LSFSSPKSESLLVDKASSNATLPFSYHQLSSYSRNTGYNSG SISMHGALA
**10**	31	2.1e-041	FAHNNVHHPLPPHSSPANTINRMFTSNKENN
