# Supplementary material for: Genome-wide identification of GRF transcription factors in soybean and expression analysis of GmGRF family under shade stress
Source: BMC Plant Biol. 2019 Jun 21;19:269. doi: 10.1186/s12870-019-1861-4 (PMC6588917; doi:10.1186/s12870-019-1861-4)
Supplement: Supplementary file 3 — Table S2. Duplication events of GmGRFs. (PDF 44 kb) [file 12870_2019_1861_MOESM3_ESM.pdf]

Additional file 3: Table S2. Duplication events of *GmGRFs*.

Segment pairs	Duplication Type	Ka	Ks	Ka/Ks
GmGRF1* & *GmGRF8	WGD or segmental duplication	0.01707	0.10052	0.16980
GmGRF2* & *GmGRF10	WGD or segmental duplication	0.02404	0.05738	0.41902
GmGRF2* & *GmGRF11	WGD or segmental duplication	0.14613	0.50439	0.28971
GmGRF2* & *GmGRF13	WGD or segmental duplication	0.59754	1.50426	0.39723
GmGRF3* & *GmGRF9	WGD or segmental duplication	0.97777	1.07045	0.91343
GmGRF3* & *GmGRF20	WGD or segmental duplication	0.04917	0.14414	0.34109
GmGRF4* & *GmGRF5	WGD or segmental duplication	0.98223	1.05698	0.92927
GmGRF6* & *GmGRF7	WGD or segmental duplication	0.49383	1.07872	0.45779
GmGRF6* & *GmGRF14	WGD or segmental duplication	0.69148	0.92171	0.75022
GmGRF6* & *GmGRF15	WGD or segmental duplication	0.84738	1.41322	0.59961
GmGRF6* & *GmGRF16	WGD or segmental duplication	0.01202	0.09606	0.12518
GmGRF6* & *GmGRF17	WGD or segmental duplication	0.97983	1.06030	0.92410
GmGRF7* & *GmGRF14	WGD or segmental duplication	0.22864	0.23588	0.96933
GmGRF7* & *GmGRF15	WGD or segmental duplication	0.01341	0.10911	0.12286
GmGRF7* & *GmGRF16	WGD or segmental duplication	0.51802	1.04386	0.49625
GmGRF7* & *GmGRF17	WGD or segmental duplication	0.23990	0.23990	0.99997
GmGRF9* & *GmGRF20	WGD or segmental duplication	0.78413	0.72390	1.08321
GmGRF10* & *GmGRF11	WGD or segmental duplication	0.12365	0.53004	0.23328
GmGRF10* & *GmGRF13	WGD or segmental duplication	0.18986	0.09688	1.95973
GmGRF14* & *GmGRF15	WGD or segmental duplication	0.16360	0.47851	0.34189
GmGRF14* & *GmGRF16	WGD or segmental duplication	1.05567	0.84077	1.25561
GmGRF14* & *GmGRF17	WGD or segmental duplication	0.36359	0.27617	1.31652
GmGRF15* & *GmGRF16	WGD or segmental duplication	0.52220	0.95654	0.54593
GmGRF15* & *GmGRF17	WGD or segmental duplication	0.71435	1.17212	0.60946
GmGRF16* & *GmGRF17	WGD or segmental duplication	0.70230	0.81631	0.86033
GmGRF18* & *GmGRF21	WGD or segmental duplication	0.03507	0.16629	0.21089
GmGRF18* & *GmGRF19	Tandem duplication	0.32966	0.47921	0.68792
GmGRF21* & *GmGRF22	Tandem duplication	0.21642	0.33387	0.64822
