# Supplementary figures and images for: Genome-wide identification of GRF transcription factors in soybean and expression analysis of GmGRF family under shade stress
Source: BMC Plant Biol. 2019 Jun 21;19:269. doi: 10.1186/s12870-019-1861-4 (PMC6588917; doi:10.1186/s12870-019-1861-4)

**Additional file 4: Figure S2**

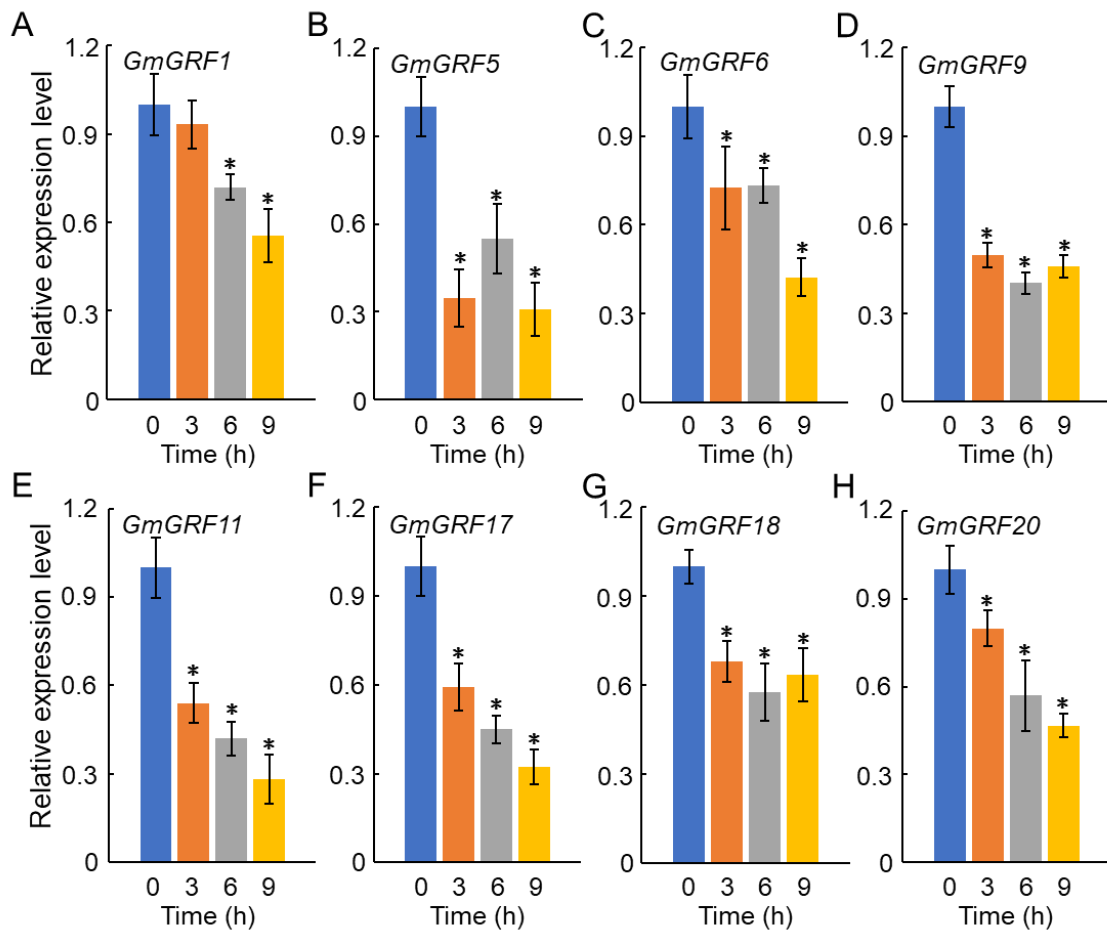

Supplement: Supplementary file 4 — Figure S2. The transcription levels of GmGRFs in response to GA3. The housekeeping GmTubulin was used as an internal control. Error bars represent standard errors. The asterisk (*) indicates the significant difference at P < 0.05 by Student’s t-test analysis. (PDF 46 kb) [file 12870_2019_1861_MOESM4_ESM.pdf]

Additional file 5: Figure S3

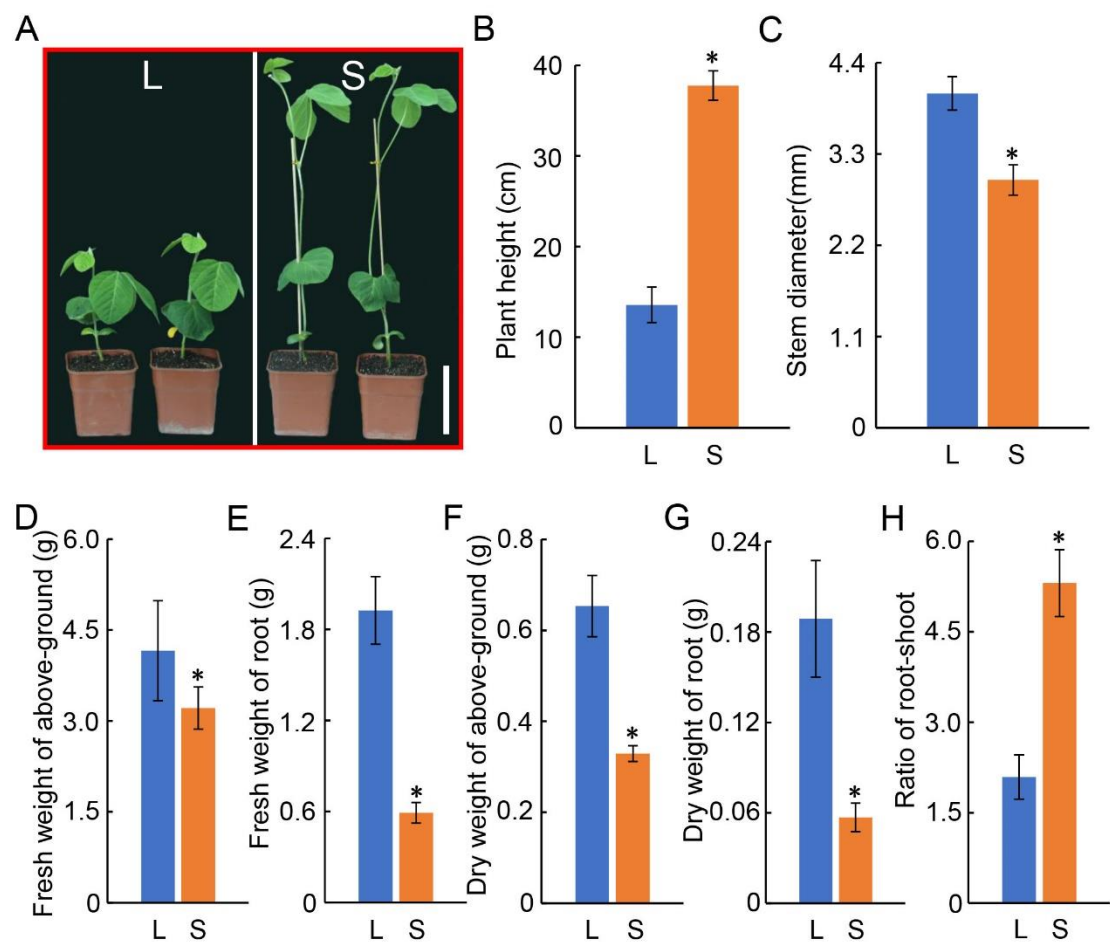

Supplement: Supplementary file 5 — Figure S3. Shade stress induces shade avoidance response in soybean. (A) Representative photographs of soybean seedlings under white light and shade conditions. Bar = 100 mm. Seeding height (B), stem diameter (C), above-ground tissue fresh and dry weight (D, F), root fresh and dry weight (E, G) and root-shoot ratio (H). Ten soybean seedlings were measured under each condition. Error bars represent standard errors. The asterisk (*) indicates the significant difference at P < 0.05 by Student’s t-test analysis. L, shade; S, shade. (PDF 134 kb) [file 12870_2019_1861_MOESM5_ESM.pdf]
