# Supplementary material for: Genome-wide identification of GRF transcription factors in soybean and expression analysis of GmGRF family under shade stress
Source: BMC Plant Biol. 2019 Jun 21;19:269. doi: 10.1186/s12870-019-1861-4 (PMC6588917; doi:10.1186/s12870-019-1861-4)
Supplement: Supplementary file 6 — Table S3. Genome size and GRF number in plants. (PDF 32 kb) [file 12870_2019_1861_MOESM6_ESM.pdf]

Additional file 6: Table S3. Genome size and *GRF* number in plants.

**Plants**	**Scientific name**	**Number of *GRF* genes**	**Genome size (Mb)**
Arabidopsis	*Arabidopsis thaliana*	9	125
Rice	*Oryza sativa*	12	466
Maize	*Zea mays*	14	2300
Cabbage	*Brassica rapa*	17	485
Brachypodium	*Brachypodium distachyon*	10	260
Cucumber	*Cucumis sativus*	8	350
Melon	*Cucumis melo*	8	450
Watermelon	*Citrullus lanatus*	8	425
Poplar	*Populus trichocarpa*	19	480
Pear	*Pyrus bretschneideri*	10	527
Grape	*Vitis vinifera*	8	490
Tomato	*Solanum lycopersicum*	13	900
Tea tree	*Camellia sinensis*	6	3100
Sweet orange	*Citrus sinensis*	9	367
Tobacco	*Nicotiana tabacum*	25	4410
Oilseed rape	*Brassica napus*	35	630
Common bean	*Phaseolus vulgaris*	10	587
Soybean	*Glycine max*	22	1100
Medicago	*Medicago truncatula*	8	500
