# Supplementary material for: Genome-wide identification of GRF transcription factors in soybean and expression analysis of GmGRF family under shade stress
Source: BMC Plant Biol. 2019 Jun 21;19:269. doi: 10.1186/s12870-019-1861-4 (PMC6588917; doi:10.1186/s12870-019-1861-4)
Supplement: Supplementary file 7 — Table S4. MtGRFs in Medicago. (PDF 20 kb) [file 12870_2019_1861_MOESM7_ESM.pdf]

Additional file 7: Table S4. *GRFs* in *Medicago*.

Serial No.	Name	Gene
**1**	***MtGRF1***	MTR_1g017490
**2**	***MtGRF2***	MTR_2g041430
**3**	***MtGRF3***	MTR_3g092330
**4**	***MtGRF4***	MTR_4g125490
**5**	***MtGRF5***	MTR_5g027030
**6**	***MtGRF6***	MTR_7g104050
**7**	***MtGRF7***	MTR_8g020560
**8**	***MtGRF8***	MTR_0001s0490
