# Supplementary material for: Genome-wide identification of GRF transcription factors in soybean and expression analysis of GmGRF family under shade stress
Source: BMC Plant Biol. 2019 Jun 21;19:269. doi: 10.1186/s12870-019-1861-4 (PMC6588917; doi:10.1186/s12870-019-1861-4)
Supplement: Supplementary file 8 — Table S5. Primers sequence used in this study. (PDF 40 kb) [file 12870_2019_1861_MOESM8_ESM.pdf]

Additional file 8: Table S5. Primers sequence used in this study.

Gene	Forward primer	Reverse primer
GmTubulin	AACCTCCTCCTCATCGTACT	GACAGCATCAGCCATGTTCA
GmGRF1	ATGTCTCATCCATCTCTGGGGT	TCGCAGTACTTGGAGTCAGG
GmGRF5	AACCCCCTTCAGCAGGTGTG	CATGTGGAACAGGAACAGAAGC
GmGRF6	AGAAGCTGAGACAGCTACGG	CGGTTTGGATTCTGGAGTTGG
GmGRF9	GGGATGGCAAATCAAATGGC	TGTCAGGCTCTTGCAGTGTC
GmGRF11	GTCTCAGTGGCAGGAACTGG	CGTGCGAGAGCGAGATAGTAG
GmGRF17	CCCTCTTCCGGTGGTGTTTC	AGAGCATGAAGCAGTGTCGAG
GmGRF18	GAGGACGCCATCGTTCAAGA	CCAGGCACCACAGAGTTGG
GmGRF20	CAATGCCCGGAAATGCTGCT	GGCCTGGGTCCTCTCCATAG
